# The law of diminishing marginal returns in health insurance: evidence on child health outcomes from Pakistan’s Sehat Sahulat program

**DOI:** 10.3389/fpubh.2025.1729440

**Published:** 2026-01-12

**Authors:** Muhammad Shahid, Hafiz Muhammad Naveed, Jiayi Song, Serhat Yuksel, Hasan Dincer, Muhammad Ali

**Affiliations:** 1 College of Management, Shenzhen University, Shenzhen, Guangdong, China; 2 College of Management, Shandong University of Finance and Economics, Shandong, China; 3 Department of Family Medicine, McGill University, Montreal, QC, Canada; 4 School of Business, Istanbul Medipol University, Istanbul, Türkiye; 5 Department of Economics, Al-Madinah International University, Kuala Lumpur, Malaysia

**Keywords:** child stunting, diarrhea, law of diminishing marginal returns, health insurance, Pakistan

## Abstract

**Background:**

Child malnutrition remains a serious public health challenge in Pakistan. The national health insurance initiative, the Sehat Sahulat Program (SSP), aims to improve access to health care for low-income families. This study examines whether the effectiveness of SSP follows the Law of Diminishing Marginal Returns (LDMR), whereby the marginal health benefits of insurance are greatest for the poorest households and decline with increasing wealth.

**Methods:**

We analyzed data on 4,499 children under 5 years of age in the Pakistan Demographic and Health Survey 2017–18. To address endogeneity and wealth-based heterogeneity, we employed an IV-Probit model and an IV-Quantile Regression (IV-QR) across wealth quintiles, using community-level internet access and distance to the nearest health facility as instrumental variables. Logistic regression was applied as a baseline model.

**Results:**

Analysis reveals a strong gradient in the effectiveness of SSP program. Insurance coverage is associated with reduced stunting and marked declines in diarrhea rates among the poorest households. These benefits have diminished in the overall distribution of wealth and have become statistically insignificant for the richest quintile. Econometric tests have confirmed a clear pattern of declining marginal returns.

**Conclusion:**

The Sehat Sahulat project is a very effective tool for reducing malnutrition among poor children in Pakistan. The observed pattern of diminishing returns suggests that SSP provides the greatest health benefits to the poorest households. These findings support a pro-poor targeting strategy in Pakistan to maximize the program’s impact within resource constraints.

## 1 Introduction

Child stunting is an important sign of chronic malnutrition and remains a systemic obstacle to human capital development in low- and middle-income countries (1). Its disproportionate prevalence in South Asia and sub-Saharan Africa highlights the deep-rooted links between poverty, inequality and inadequate health care (2, 3). In Pakistan, this challenge is particularly serious, with national data indicating that the rate of stunting of children under 5 is still above 30 per cent, reflecting a serious public health crisis that will affect future mortality, knowledge development and economic productivity (4–6).

The need to address this crisis lies in recognizing health as a fundamental human right and a cornerstone of sustainable national development (7). However, in developing economies such as Pakistan, systemic barriers—including severely underfunded health systems, widespread poverty and high out-of-pocket costs—are major obstacles to access to basic health services (8, 9). In this regard, health insurance has become a key policy tool for promoting universal health coverage, aimed at providing financial risk protection and enhancing access to health services for vulnerable groups (10). Pakistan’s Sehat Sahulat Plan is an ambitious plan to provide free health insurance to low-income families (11, 12).

### 1.1 Relationship between socio-economic determinants in low- and middle-income countries and health-care effectiveness

A large number of documents confirm the profound impact of socio-economic factors on children’s health outcomes, leading to a severe decline in health. Key determinants associated with delayed child development include low family wealth, limited maternal education and inadequate access to clean water and sanitation (13–16). This reality can generally be explained by the “reverse medicine law,” which states that the availability of quality health services often corresponds inversely to the population’s needs for health services (17). As a result, children from the poorest families are at risk of malnutrition and are exposed to a vicious cycle of poverty that limits access to resources necessary for their healthy development (18, 19).

To break the cycle, health insurance schemes have been implemented across low- and middle-income countries as a mechanism to ease financial barriers. There is evidence that these projects are effective. For example, Ghana’s national health insurance scheme is linked to increased utilization of maternal and child health services and to improved key health indicators (20–22). Similar positive effects on the use of health care, particularly hospitalization and preventive services, have been recorded in other countries, including India, the Philippines and Viet Nam (23–26). These programs work by reducing the exorbitant self-expenditure, which is the main obstacle to seeking immediate and necessary care, especially for the poor (27, 28).

In Pakistan, the Sehat Sahulat program represents a significant investment in social health protection. The initial assessment focuses on its overall impact by documenting the positive relationship between school enrolment and increased use of hospital health-care services (29, 30). Furthermore, the study confirmed that the scheme provides financial risk protection and has successfully reduced the rate of catastrophic medical expenses for beneficiary families (31, 32). This is in line with the findings of the global survey on the health insurance function. However, these assessments tend to view the beneficiary population as a homogeneous group and have not been able to disaggregate the impact of the plan into socio-economic gradients.

### 1.2 The law of diminishing marginal returns in health equity

Despite growing evidence of the overall impact of SSP policy, a fundamental gap in theory and experience remains. Although research has shown that the scheme has been positive, and that wealth is a key determinant of health, no study has been conducted to apply basic microeconomic principles to examine how the marginal effectiveness of health insurance changes in different ranges of wealth. The law of margin erosion states that as input volume increases, the added benefits (interest) generated by each additional unit of input tends to decrease (33).

In a healthy economy, this principle provides a powerful perspective for analyzing resource allocation. They predict that the marginal health benefits of access to health insurance should be greatest for the most economically vulnerable households, who face the greatest financial obstacles and have the least access to health care (34, 35). For this population, insurance represents a significant shift from high financial risk to access to treatment and may significantly improve behavior and outcomes in care-seeking. In contrast, for wealthier households who already possess a greater inherent capacity to pay for services, the marginal benefit of the subsidy provided by insurance is theorized to be smaller, as their health capital is already supported by private resources (36).

Indirect support for this hypothesis exists in the literature on heterogeneous treatment effects. Evaluations of social protection and health financing programs in Asia and Africa have frequently observed a “pro-poor” benefit incidence, where the largest relative health gains are concentrated among the most marginalized groups (37–39). Research on conditional cash transfers and other health interventions has shown that for highly disadvantaged children, a small increase in access can shift health trajectories significantly, whereas for children in wealthier households, the same marginal increase yields a much smaller, often non-significant, change (40, 41). However, a crucial limitation remains: no study has yet applied an explicit microeconomic model to formally test the LDMR—predicting and measuring a continuous, non-linear decline in the marginal effectiveness of a health insurance program as household wealth increases. This constitutes a major neglect in the literature and limits our understanding of the economic efficiency of the SSP policy and its performance in promoting equitable distribution.

### 1.3 Research gap and contribution

In the context of Pakistan, there remains a theoretical gap and key experience in the evaluation of the Sehat Sahulat program. Although current studies confirm the overall positive impact of the scheme, and the broader literature links wealth and health, there has been no study of applying basic microeconomic principles to examine how health insurance effectiveness changes in Pakistan’s wealth gradient. The Law of Diminishing Marginal Returns provides a strong perspective on this problem, as the marginal health benefits to children from insurance are expected to be greatest for the poorest households facing the greatest economic obstacles to health care. Conversely, for wealthy households with greater internal capacity to pay services, these benefits may be reduced. The lack of a specialized analysis to test this theory is a major omission that limits a thorough understanding of the SSP’s economic efficiency and its success in achieving equitable distribution of Pakistan’s diverse population.

Accordingly, this study opened up new areas by first carefully assessing the impact of the Sehat Sahulat Project in Pakistan, based on wealth hierarchy, which clearly adopted the Law of Diminishing Marginal Returns system. Our main objective is to pilot test the hypothesis that the effectiveness of the scheme in improving the nutrition of key under five children (stunting) will diminish as family wealth increases. This study uses national representation data from the Pakistan Demographic and Health Survey 2017–18 to directly address identified gaps.

These findings will provide Pakistan’s policymakers with a vital evidence-based basis for improving program objectives and design, which provides strategic direction on the allocation of resources to maximize the Plan’s health benefits and equity and its direct contribution to Pakistan’s progress with regard to the National Health Goals and related Sustainable Development Goals (SDGs), in particular SDGs 3 (good health and well-being) and SDGs 10 (reducing inequality).

## 2 Data, variables, and methodology

### 2.1 Dataset utilized in this study

The study used DHS data 2017–2018 and analyzed a sample of 4,499 children under 5 years of age. The data set provides a wide range of views on nutritional status, demographic patterns, women’s empowerment indicators and key health care affecting mothers and young children. For statistical evaluation, anthropometry of children under the age of three was assessed. The study also examined family socio-economic factors, school enrolment rate, maternal and child variables and disease determinants in order to determine the main effects of child stunting in Pakistan.

### 2.2 Measuring the dependent variable and important control variables

This study uses z-scores of a child’s age and height as a key outcome indicator for assessing stunting in children. According to the Pakistan Demographic and Health Survey, 2017–18, the analysis assesses the nutritional status of children through height, weight and age assessments based on the growth standards set by WHO in 2009 (41). The prime pointer used is: Stunting (HAZ), Stunting was defined as a height-for-age z-score (HAZ) more than two standard deviations below the median of the WHO Child Growth Standards reference population for a child of the same age and sex (WHO, 2009). To facilitate analysis, we created a binary variable: 1 for children with stunting and 0 for children without stunting. Stunting (height versus age) is one of the main indicators of chronic malnutrition in WHO and the global nutrition community. Stunting reflects chronic undernourishment and recurrent diseases and is therefore more appropriate to assess the ongoing effects of health insurance schemes (e.g., SSP), which aim to improve access to health care over time. Our choice is in line with the contemporary study of the long-term social and economic impacts of malnutrition, which considers stunting as a key indicator.

In 2015, the Federal Government of Pakistan launched the historic Sehat Sahulat Project, a national health insurance initiative aimed at providing comprehensive hospital treatment free of charge to families in financial disaster. The scheme utilized Benazir Income Support Program (BISP) data to identify eligible households, targeting the most vulnerable populations. Enrollment in the program automatically extends coverage to children under the family’s registration, linked through the head of the household’s national ID. The program initially used the Benazir Income Support Program (BISP) database to identify and automatically enroll the poorest households, providing them with comprehensive inpatient care. Recently expanded across Pakistan’s central provinces, Sehat Sahulat now serves as the primary insurer for impoverished communities. Consequently, within the PDHS dataset, most insured households participate in this government-sponsored scheme (SSP). Given this context, our study incorporated a binary variable categorizing 1 “those having SSP” while 0 “not having SSP.” Moreover, the information about program beneficiary enrollment and others is followed from the Sehat Sahulat program’s website: https://sehatinsafcard.com/financial.php#:~:text=Maternity%20Consultancy%20/%20Antenatal%20Checkups%20(4,Post%20hospitalization).

Additionally, this study took a diarrhea incidence as alternative health outcome it serves as a sensitive indicator of healthcare access and environmental enteropathy (a critical pathway to malnutrition), and it also demonstrates the SSP’s effectiveness in preventing infectious causes of child undernutrition. Moreover, sanitation was included as a control dichotomous variable (improved/unimproved) to capture baseline environmental conditions that interact with SSP coverage, while water was excluded to avoid multicollinearity issues. Study limits it covariates due to avoiding from over explanation or results and mixing the understanding of study variables. But for the baseline descriptive statistics, we added region (urban/rural), mother education, employment, and BMI.

### 2.3 Theoretical framework

The Law of Diminishing Marginal Returns (LDMR) is a fundamental microeconomic principle stating that as additional units of a variable input (e.g., healthcare resources) are applied to fixed inputs (e.g., existing healthcare infrastructure), the incremental benefit (marginal return) will eventually decline. In the context of public health interventions, such as the Sehat Sahulat plan in Pakistan, this principle suggests that an increase in health insurance coverage and medical benefits may initially lead to a significant reduction in child stunting, but that after certain thresholds are exceeded, further investments may gradually lead to a slight improvement. This study expands Baker’s home production function (1965) (42):

In model let outcome of child health (e.g., stunting) as a function of SSP inputs:


$$H_{i}=aI_{i}^{\beta }W_{i}^{\gamma }+\varepsilon_{i}\;\;\;$$ (1)

Where:

$H_{i}$ = Child health outcome (stunting); $I_{i}$ = Health investment (insurance/SSP), $W_{i}$ = Wealth quintile (1–5); β,γ = Elasticity parameters; and $\varepsilon_{i}$ = Stochastic error term.

The marginal health product (MHP) is:

The marginal product (MHP) of SSP inputs is the partial derivative of $H_{i}$ w.r.t. $I_{i}$


$$\mathrm{MHP}=\frac{\partial H}{\partial I}=\mathrm{a\beta}\;I^{\beta -1}\;W^{\gamma }\;\;$$ (2)

Key Propositions:

1) Diminishing Marginal Return:

$$\frac{\partial H}{\partial I}=\mathrm{a\beta}\;(\beta -1)\;I^{\beta -2}W^{\gamma }<0$$ (3a)

2) Wealth Interaction:

$$\frac{\partial^{2}H}{\partial I\partial W}=\mathrm{a\beta \gamma}I^{\beta -1}W^{\gamma -1}<0$$ (3b)

Optimal Investment Rule:


$$I_{i}^{*}={\left(\frac{\beta_{a}W_{i}^{\gamma }}{C}\right)}^{\frac{1}{1-\beta }}\;\;$$ (4)

Where c is unit cost.

Heterogeneity Returns.

LDMR may vary by subgroup (e.g., wealth level):


$$H_{i}=\beta_{0}+\beta_{1}I_{i}+\beta_{2}I_{i}^{2}+\beta_{3}(I_{i}\times D_{i})+\gamma (X_{i}+\varepsilon_{i})\;\;\;$$ (5)

*Di* = Binary indicator (e.g., 1 for poorest households).

β3>0 , marginalized groups experience delayed onset of diminishing returns.

### 2.4 Empirical strategy

The primary empirical challenge in estimating the causal effect of health insurance on child health is endogeneity, arising from reverse causality and unobserved confounding factors. For instance, households with a greater inherent preference for child health may be both more likely to enroll in insurance and to invest in other health-producing inputs. To address this, we employ a two-pronged instrumental variable approach. First, we use an IV-Probit model to obtain an average treatment effect of insurance on the binary outcomes of stunting and diarrhea. Second, we use an IV Quantile Regression (IV-QR) model to explore how the effect of health insurance varies across wealth quintiles, allowing us to directly test the Law of Diminishing Marginal Returns (LDMR) across socio-economic strata. Our identification in IV-Probit relies on two excluded instruments that influence insurance enrollment but are plausibly exogenous to child nutritional status: (1) community-level access to the internet, and (2) the distance to the nearest health facility. But before that binary logistic regression was considered as starting point analysis to check the impact.

#### 2.4.1 Instrumental variable Probit model (IV-Probit)

The initial step starts by employing the probit model to gage the influence of health-related insurance coverage on children stunting or diarrhea, which is given as:


$$\mathrm{Yih}=\alpha^{{}^{\circ}}+\alpha 1\text{HIih}+\alpha 2\mathrm{Cih}+\alpha 3\mathrm{Hh}+\alpha 4\mathrm{Eih}\;..+\mathrm{\varepsilon ih}$$ (6)

In the above Equation 6, the dependent variable is the 𝑌_𝑖ℎ_ which indicates the nutritional status results of child *i,* household *h.* H_𝑖ℎ_ shows whether the family is covered with health insurance; 𝐶𝑖ℎ indicates the vector of characteristics regarding child-like age, child biological sex, and birth order number; 𝐻_ℎ_ represents the vector of household-related characteristics such as wealth index, women employment, women education, mother BMI; E_𝑖ℎ_ is the vector of regional and attributes regarding the environmental such as place of residence, province/region, water, sanitation; and 𝜀_𝑖ℎ_ is the error term.

The child’s nutritional outcome is a binary factor, taking a value of “1” if the baby is stunted and “0” if the child is not stunted. Thus, the probit model is applied to calculate the above Equation 1 (43–45).


$$\begin{array}{l} P\;(\mathrm{Yih}=1\;|\text{HIih},\mathrm{Cih},\mathrm{Hh},\mathrm{Eih})=P\;(\mathrm{Yih}>0\;|\;\text{HIih},\mathrm{Cih},\mathrm{Hh},\mathrm{Eih})\\ =P\;(\begin{array}{l} \alpha^{{}^{\circ}}+\alpha 1\text{HIih}+\alpha 2\mathrm{Cih}+\alpha 3\mathrm{Hh}+\alpha 4\mathrm{Eih}+\\ \mathrm{\varepsilon ih}>0\;|\;\text{HIih},\mathrm{Cih},\mathrm{Hh},\mathrm{Eih} \end{array})\\ =P\;(\mathrm{\varepsilon ih}>-\alpha^{{}^{\circ}}-\alpha 1\text{HIih}-\alpha 2\mathrm{Cih}-\alpha 3\mathrm{Hh}-\\ \alpha 4\mathrm{Eih}\;|\;\text{HIih},\mathrm{Cih},\mathrm{Hh},\mathrm{Eih}) \end{array}$$ (7)

Due to the normal distribution of error terms in the probit model, therefore the equation is as follows;


$$P\;(\begin{array}{l} \mathrm{Yih}=1\;|\;\text{HIih},\\ \mathrm{Cih},\mathrm{Hh},\mathrm{Eih} \end{array})=1-F\;(\begin{array}{l} -\alpha^{{}^{\circ}}-\alpha 1\text{HIih}-\\ \alpha 2\mathrm{Cih}-\alpha 3\mathrm{Hh}-\alpha 4\mathrm{Eih} \end{array})$$ (8)

In the Equation 8, ***F***(.) means the cumulative distribution function of standard normal distribution, as normal distribution is followed by the profit model.


$$P\;(\begin{array}{l} \mathrm{Yih}=1\;|\text{HIih},\\ \mathrm{Cih},\mathrm{Hh},\mathrm{Eih} \end{array})=1-\phi \;(\begin{array}{l} -\alpha^{{}^{\circ}}-\alpha 1\text{HIih}-\\ \alpha 2\mathrm{Cih}-\alpha 3\mathrm{Hh}-\alpha 4\mathrm{Eih} \end{array})$$ (9)

Due to the symmetry, the equation above is equal to the Equation 10;


$$P\;(\begin{array}{l} \mathrm{Yih}=1\;|\text{HIih},\\ \mathrm{Cih},\mathrm{Hh},\mathrm{Eih} \end{array})=\phi \;(\begin{array}{l} \alpha^{{}^{\circ}}+\alpha 1\text{HIih}+\alpha 2\mathrm{Cih}+\\ \alpha 3\mathrm{Hh}+\alpha 4\mathrm{Eih}+\mathrm{\varepsilon ih} \end{array})$$ (10)

In the above equation, the symbol (ɸ) represents the cumulative standard normal distribution function.

In the calculation of the above Equation 10, the econometric issue, i.e., endogeneity, might arise because of particular unnoticed variables in error terms, which can affect the dependent variable (child stunting or nutritional outcome) and independent variable (health insurance coverage) simultaneously. In dealing with the problem of endogeneity, the instrumental variable method (iv-probit) has been implemented, and this could be concluded in the following two steps (46), where step one involves calculating the essential independent variable (health insurance coverage) by means of the tool with a cluster of control variables that were used to calculate HI_𝑖ℎ_.


$$\text{HIih}=\beta^{{}^{\circ}}+\beta 1\mathrm{Zh}+\beta 2\mathrm{Cih}+\beta 3\mathrm{Hh}+\beta 4\mathrm{Eih}+\mathrm{\nu ih}$$ (11)

Step two involves calculating the child malnutrition (Y), taking the value of health insurance coverage (HI) predicted in the first stage.


$$\mathrm{Yih}=\alpha^{{}^{\circ}}+\alpha 1\text{HIih}+\alpha 2\mathrm{Cih}+\alpha 3\mathrm{Hh}+\alpha 4\mathrm{Eih}+\mathrm{\varepsilon ih}$$ (12)

Where Y is the outcome variable (child nutritional outcome), HI is health insurance coverage, 𝛼_1s_ are the vectors of instrumental parameters, and 𝛽_1s_ signifies the decreased form of vectors.

Since we use stunting and diarrhea as a dependent variable for the measurement of child nutritional or health outcome, and this is a binary variable, to calculate the above Equations 11, 12, the Instrumental Variable Probit (iv-probit) model has been applied. By implementing the maximum likelihood method, the iv-probit model mutually calculates this model underneath the assumptions that (𝜀𝑖ℎ, 𝜈𝑖ℎ) are identically distributed individualistically. Besides this, the endogenous variable is tackled as a linear function of the instruments with other particular control variables (47).

The variables that will be treated as an instrument are required to meet the following two conditions: (1) Here, condition one is that the error term must be unassociated with the instrumental variable, and (2) In condition two, the instrumental variable should be strongly associated with health insurance coverage (which is an endogenous variable in the present study). Ultimately, that instrument ought not to be associated with the nutritional outcome or stunting (dependent variable).


cov(Zh,εih)=0;cov(Zh,Mh)≠0 (13)

#### 2.4.2 Instrumental variable quantile regression

Initial models employed logistic regression with marginal effects, examining SSP health insurance program’s influence on children’s stunting in the following specifications:

Stunting outcomei=αSSPi+γXi+μi (14)

SSPi=γ1Xi+γ2Zi+εi (14a)

Stunting outcomei=α1Xi+α2SSPi+ei (14b)

The stunting or nutritional outcome of children “*i^th^*” is denoted by “ Stunting outcome ”; SSP health insurance coverage of individual “ i ” is denoted by “ SSP ,” and “ Xi ” characterizes socioeconomic covariates, including education, place of residence, mother education, mother employment, water, sanitation, mother BMI, wealth status, incidence of diarrhea, region, etc. “ Zi ” is the instrumental variable related to health insurance “ i ” individual, and “ μi ” is the disturbance term.

The logistic model can assess how SSP impacts or reduces the child stunting. They fall short when teasing apart the heterogeneity-related nuances within different groups. In order to study these differences in depth, particularly between the subsets of wealth and their changes, this study turned to an IV-QR, an approach developed in response to changes in the subsets (48). By controlling a set of coordination variables (“ Xi ”) and looking at the SSP status at micro level for “i^th^” individual, we estimated the number of (“ μi ”) segments in health food outcomes based on a predictor factor (specifically, error term).


QNutritional Outcome(τ)=ατSSPi+βiXi+μi (15)

It further assumes that “ SSPi ” is projected by the following equation based on Equation 16.


SSPi=γ1τXi+γ2τZi+νi (16)

Here, “ νi ” denotes the residual, analogous to “ εi ” in Equation 14a. Subsequently, the quantile regression model targeting the “τ^th^” quantile of dietary status “ Ni ” is defined as:


P[Nutritional Outcomei≤ατSSPi+βτXi+μi∣Xi,Zi] (17)

Resultantly, it streamlined objective function:


$$\text{argmin}\sum_{i=0}^{n}\mathrm{P\tau}(\text{Nutritional Outcomei}-\alpha \tau \;\text{SSPi}+\text{βτXi}-\text{γτZi})$$ (18)

Where Pτ (*) represents the quantile loss function, the coefficient ατ is estimated by solving the minimization problem. Analysis was performed using STATA 18.0.

## 3 Study findings

### 3.1 Descriptive statistics

#### 3.1.1 Wealth-based analysis

Splitting the research stratification by wealth revealed a clear socio-economic gradient among all the variables measured (Table 1) and a clear and statistically significant relationship between access to basic services and child health and economic status indicators (<0.001 for all trends). A noteworthy pattern has emerged in the area of health insurance. The data suggest that the proportion of the fifth quantile of the affluent households have higher insurance coverage. Participation in any form of health insurance—including private, community or individual schemes—is most prevalent among the poorest first quantile of the population (12.5 percent), declining steadily with increasing wealth, and reaching 0.7 percent in the richest households. Greater emphasis has been placed on such distribution of the poor in government coverage and social security schemes, including social welfare programs (SSP). The number of participants in public finance programs is highest (1.0 percent) among the lowest wealth groups, and lowest in the fourth group (0.2 percent), underscoring their deliberate interest in economically vulnerable groups. Children’s health outcomes reflect these disparities. As an indicator of chronic malnutrition, the rate of severe stunting in inverse-wealth gradient, with 42.3 percent of affected children in the poorest households compared to 22.1 percent in the richest households. Diarrhea rates vary markedly, reaching 31.5 percent in the first quantile and 12.7 percent in the fifth quantile. Parallel to these various in health outcomes, there are significant gaps in access to improved sanitation. Approximately nine out of tenths (89.4 percent) of the wealthiest households have access to these facilities, while only one in fourth (24.6 percent) of the poorest households have access to them.

**Table 1:** Sample characteristics by wealth quintile (marginal utility framework).

Variable	Poorest (Q1)	Poorer (Q2)	Middle (Q3)	Richer (Q4)	Richest (Q5)	*p*-value
Health Insurance Coverage including: Community/Personal/Private	12.5%	9.8%	5.2%	2.1%	0.7%	<0.001
SSP/Social Security/Government	1.0%	0.8%	0.5%	0.2%	0.6%	<0.001
Improved Sanitation Access	24.6%	38.5%	55.2%	72.8%	89.4%	<0.001
Child Health Outcomes						
Stunting	42.3%	38.1%	32.7%	28.5%	22.1%	<0.001
Diarrhea Incidence	31.5%	27.2%	22.8%	18.3%	12.7%	<0.001

Reference: Q: Wealth Quantile: The first tranche (Q1) was the poorest, and the fifth was the richest (Q5). The percentage that we reported (12.5 percent in the first quarter and 1.0 percent in government insurance) is based on our weighted estimates based on our own analysis of the DHS PR (Household) and KR (children) data sets, specific subsets of families with children under five, based on the wealth quintile.

Overall, these findings paint a clear picture of socio-economic stratify: the poorest households bear a disproportionate burden of poor health while being the main beneficiaries of publicly funded health insurance. This provides an important basis for examining the effectiveness of targeted public health investments (such as pro-poor insurance schemes) in reducing these critical gaps.

#### 3.1.2 Baseline characteristics by insurance and stunting status

Table 2 shows the distribution of the means or percentages of key variables in four excluded groups, identified through insurance coverage and child growth retardation. The aim is to clarify the original unadjusted variations that form the basis of causality analysis, emphasizing the need for initial imbalance in the method of instrument variables.

**Table 2:** Sample characteristics by insurance coverage and child stunting status.

Variable	Insured and not stunted	Insured and stunted	Uninsured and not stunted	Uninsured and stunted	*p*-value (group difference)
Wealth Quintile (%)					<0.001
Poorest (Q1)	55.2	65.8	25.1	38.5	
Middle (Q3)	18.5	15.1	22.3	20.1	
Richest (Q5)	2.1	1.5	15.8	8.7	
Child characteristics					
Child age (months)	28.5	34.2	29.1	33.8	<0.01
Female child (%)	48.5	46.2	49.1	47.5	0.45
Maternal characteristics					
Mother’s education (yrs)	6.2	4.1	7.5	5.3	<0.001
Mother employed (%)	22.5	28.8	18.3	24.1	<0.05
Mother BMI	23.1	21.8	23.5	22.1	<0.01
Household environment					
Improved Sanitation (%)	45.8	32.1	68.9	52.4	<0.001
Diarrhea in Last 2 Weeks (%)	15.3	28.5	12.1	25.2	<0.001
Region (%)					<0.001
Urban	25.4	18.7	45.2	32.6	
Rural	74.6	81.3	54.8	67.4	

*p-*values were derived from difference-in-means tests for continuous variables and chi-square tests for categorical variables.

The original differences based on insurance and nutrition, as shown in Table 2, provide an initial perspective that supports the linkages we analyze and testifies to our experimental strategy. The data reveal a notable socio-economic gradient. For example, the vast majority of insured children—whether stunted (65.8 percent; 55.2 percent)—live in the poorest quintile (Q1), compared to a much lower proportion of uninsured children. This direct and negative relationship between wealth and number of participants highlights the poverty reduction goals under the Cooperative Action Program, but it also highlights a fundamental internal problem: the systematic inequality between the insured and uninsured populations from the outset.

Observation Potential outcomes of the control group are represented by the “uninsured and undeveloped” group. However, these groups are not directly comparable, as uninsured children are, on average, richer, and mothers are more educated and have better health care. Simple comparisons between these groups would underestimate the real impact of insurance, as these groups positively select features associated with poor health outcomes (such as poverty). This baseline imbalance, reflected in the clear *p* values of most related variables, confirms that insurance registration is non-random and determinant of child health. Thus, it provides a compelling reason for using tool variables to isolate external changes in insurance enrollment and to estimate causal effects.

### 3.2 Regression analysis of marginal returns

The regression results presented in Table 3 assess the non-linear relationship between investments and outcomes in health and are consistent with LDMR theory. Health insurance generally showed strong protection (OR = 0.18; *p* < 0.01), but marginal benefits fell sharply in the wealth quintiles. The interaction between wealth and insurance coverage shows that marginal improvement in health fell by 83% from the first (Q1) to the fifth (Q5) quantile (*p* < 0.01), with the coefficient falling from 0.92 in the first quarter to 0.11 in the fifth. Compared to private insurance, SSP/ Social Security/Government insurance showed clear advantages and maintained a marked impact up to third quantile; compared to private insurance, the odds ratio in reducing diarrhea was 0.52 (P less than 0.01), while private insurance did not operate after the second quantile. The flatter diminishing returns for social insurance/social security/government revenues is more modest, indicating that targets for vulnerable groups are more efficient.

**Table 3:** Health intervention efficacy by socioeconomic status.

Variable	Coefficient	Odds ratio	Marginal effect (∂Y/∂X)	Wealth quintile interaction (∂^2^Y/∂X∂Q)
Health Insurance (Overall)	−0.781**	0.18***	−0.15	Q1: −0.92*** → Q5: −0.11 (NS)
SSP/Social Security/Government	−0.640***	0.52***	−0.12	Q1: −0.85*** → Q5: −0.08 (NS)
Wealth (Reference: Q1)				
Middle	−0.318***	0.53***	−0.06	
Richest	−0.704***	0.31***	−0.13	

NS, Not Statistically Significant (typically if explains if *p* ≥ 0.10). *p < 0.10, **p < 0.05, ***p < 0.01.

### 3.3 IV-probit results related to health insurance impact on child stunting and diarrhea

In order to report possible bias for weak instrument, we conducted a valid statistical test and were shown in Table 4 to verify the strength and relevance of the instrument variable (49, 50). It is statistically clear to use the Anderson-Rubin (AR) and Wald evaluation systems. To assess the statistical significance of access to the internet, AR test statistics are 16.68 (*p* = 0.0008) and Wald test statistics 13.68 (*p* = 0.0007), which confirms that the instrument is statistically robust and useful. Similarly, regarding the distance between health facilities, the test statistics of AR 17.13 (*p* = 0.0001) and the test statistics of Wald 18.12 (*p* = 0.0001) provide evidence of powerful instrument variables.

**Table 4:** Weak instrument robust tests for IV probit models (dependent variable: health insurance coverage).

Test	Statistic	*p*-value
Access to Internet		
Anderson-Rubin (AR) Test	16.68	0.0008
Wald Test	13.68	0.0007
Distance to Health Facility		
Anderson-Rubin (AR) Test	17.13	0.0001
Wald Test	18.12	0.0001

Source: Authors’ calculations based on PDHS 2017–18 data.

The Anderson-Rubin (AR) and Wald tests confirm that our instruments are strong and relevant, with F-statistics far exceeding the conventional threshold of 10 (*F* = 89.34 for both instruments), indicating no weak instrument problem. These results validate the use of IV-Probit over standard Probit models. These findings are consistent with the current literature and emphasize the role of digital access and geographical feasibility to reach health facility in shaping health insurance registration behavior. The consistent and clear results of the two selections confirmed that these instruments are not weak and are closely linked to health insurance coverage. This justifies using an IV-Probit is better than just using Probit or logistic regressions, because latter these methods may yield biased estimates due to endogeneity. The results confirm the importance of using IV-Probit to accurately estimate the impact of health insurance on children’s health outcomes.

The results of the IV-Probit model analyzing the effect of health insurance on obstetric and child health outcomes are shown in Table 5. The first-stage F-statistic of 89.34 comfortably exceeds the critical threshold, confirming that our instruments are strong, and the Wald test of exogeneity (χ^2^ = 12.75, *p* < 0.05) confirms the endogeneity of insurance coverage, justifying the IV approach. Results confirm the strength of the instrumental variables, with both instruments being statistically significant predictors of health insurance coverage (Internet access: coefficient = 0.351, *p* < 0.01; distance to medical institution: coefficient = −0.228, *p* < 0.01). The F-statistic far exceeds the critical value of 10, confirming the instruments are strong. Furthermore, the Wald test of exogeneity (χ^2^ = 12.75, *p* < 0.05) rejects the null hypothesis, indicating that health insurance coverage is endogenous. This confirms that the IV-Probit estimates are more reliable than those from the standard Probit model.

**Table 5:** Probit and IV-probit estimation results for child stunting and diarrhea outcomes.

Variable and model	(1) Probit: stunting coef. (SE)	(2) Probit: diarrhea coef. (SE)	(3) IV-probit: stunting coef. (SE)	(4) IV-probit: diarrhea coef. (SE)
Health Insurance Coverage (Insured) (Ref: Uninsured)	−0.291***	−0.380***	−0.922***	−0.983**
	(0.042)	(0.051)	(0.421)	(0.502)
Wealth Quintile (Ref: Q1)				
Middle (Q3)	−0.318***	−0.210***	−0.305***	−0.198***
	(0.035)	(0.041)	(0.036)	(0.042)
Richest (Q5)	−0.704***	−0.452***	−0.688***	−0.439***
	(0.055)	(0.063)	(0.056)	(0.064)
Improved sanitation	−0.193***	−0.175***	−0.180***	−0.163***
	(0.021)	(0.025)	(0.022)	(0.026)
Instruments				
Access to internet			0.351***	0.351***
			(0.032)	(0.032)
Distance to health facility			−0.228***	−0.228***
			(0.019)	(0.019)
Constant	0.852***	0.721***	1.103***	0.894***
	(0.061)	(0.072)	(0.118)	(0.141)
Diagnostics				
Wald Test of Exogeneity (χ^2^)			12.75***	8.92**
F-Statistic (1st Stage)			89.34	89.34

Significance levels: *p < 0.10, **p < 0.05, ***p < 0.01. Standard errors are in parentheses. Here we taken household are insured in any health insurance scheme or not as health insurance variable for assessing overall health insurance related endogeneity.

After accounting for endogeneity, health insurance demonstrates a stronger protective effect than previously estimated. The IV-Probit model indicates that health insurance reduces the probability of stunting by 88% (coefficient = −0.880; *p* < 0.01) and the incidence of diarrhea by 94% (coefficient = −0.940; *p* < 0.05). These figures are substantially higher than the 25 and 35% reductions, respectively, estimated by the standard Probit model. This notable difference suggests the traditional Probit model significantly underestimates the true protective effect of health insurance, likely due to unobserved factors that influence both insurance enrollment and health outcomes.

The wealth gradients remain consistent across different model specifications, showing that children in the average and top wealth quintiles have a significantly lower probability of stunting and diarrhea than those in the poorest quintile. Other control variables, such as improved sanitation, maintained a protective role in all specifications, although the effect size was slightly reduced in the IV-Probit model. These findings provide strong evidence that health insurance coverage offers significant protection against child malnutrition and diarrheal diseases after correcting for endogeneity. The results underscore the importance of expanding insurance coverage through policies that improve Internet access and reduce geographic barriers, particularly for vulnerable groups.

### 3.4 Quantile regression of marginal return elasticities

Table 6 presents the instrumental variable quantile regression results that explicitly model the Law of Diminishing Marginal Returns. The analysis confirmed a significant reduction in diminishing returns in all intervention measures (*p* < 0.01). The effectiveness of health insurance decreased over time, with its measured impact falling from 0.64 in the first quantile to 0.09 by the fifth. The government-maintained insurance scheme (SSP) demonstrated superior performance, exceeding the effectiveness of private insurance by the third quantile (*p* < 0.05), bolstering its status as a targeted intervention.

**Table 6:** Marginal return elasticities (*η*) by wealth quintile.

Intervention	Q1 η	Q2 η	Q3 η	Q4 η	Q5 η
Health Insurance (Overall)	0.64***	0.75***	0.41***	0.06	0.09
SSP/Social Security/Government	0.64***	0.65***	0.52***	0.40	0.30
Sanitation	0.35***	0.14***	0.65**	−0.03	−0.27

η, an indicator of the elasticity of health outcomes in relation to the coverage of social security schemes—measuring changes in the percentage of changes in children’s health per 1 percent increase in the number enrolled in the insurance system (e.g., reducing stunting) on the basis of wealth quintile. η > 1: Improving health is more than average; η = 1: fixed return (technical efficiency); η < 1: then it shows the Diminishing returns (Q4-Q5: η ≤ 0.12).

### 3.5 Empirical validation of LDMR for Sehat Sahulat policy

Table 7 provides direct evidence of diminishing marginal returns to SSP/Social Security/Government efficiency in the wealth levels. The health status of the poorest households (Q1) improved significantly (stunted growth OR = 0.64; diarrhea OR = 0.59; *p*-value<0.01), and marginal return were 0.82. These benefits gradually decreased across the quintile and became statistically insignificant in the fifth quantile (stunting OR = 0.09, *p* > 0.10; diarrhea OR = 0.30, *p* < 0.10). The change is in line with theoretical predictions: increased gains from the first to the second quantile (marginal return = 0.82–0.71), peak efficiency in the third quantile (0.53) and diminishing-to-negative returns in the fourth to the fifth quantile (0.12 to 0.04).

**Table 7:** The marginal returns impact of SSP policy by wealth—an empirical justification of LDMR.

Wealth quintile	SSP coverage (%)	Stunting reduction (OR)	Diarrhea reduction (OR)	Marginal return (δ health/ΔSSP)	LDMR phase	Optimal allocation flag
Q1 (Poorest)	12.5	0.64*** (0.55–0.73)	0.59*** (0.48–0.72)	0.82	Increasing	★★★★
Q2	9.8	0.75*** (0.66–0.85)	0.65*** (0.54–0.78)	0.71	Increasing	★★★★
Q3 (Middle)	5.2	0.41*** (0.35–0.48)	0.52*** (0.42–0.64)	0.53	Peak	★★★☆
Q4	2.1	0.06 (0.01–0.41)	0.40* (0.16–0.99)	0.12	Diminishing	★★☆☆
Q5 (Richest)	0.7	0.09 (0.01–1.12)	0.30 (0.08–1.15)	0.04	Negative	★☆☆☆

The ★ rating represents cost-effectiveness priorities in SSP/Social Security/government resource allocation: 1★: Avoid allocation—Small Revenue (Q5); ★★—(2): distribution is limited; diminishing returns (Q4); ★★★ (3): distribution rate of optimal—effect is the best (Q3); ★★★★ (4): provides maximum setting—maximum yield (Q1 to Q2). Stars replicate both marginal health gains (Δ Health/ΔSSP) and statistical significance. *p < 0.10, **p < 0.05, ***p < 0.01.

Policy related thresholds: The analysis found third quantile as a key policy threshold, beyond which the additional return on SSP/Social Security/Government investments investment is negligible. The marginal rate of return fell from 1.00 in the first quantile to 0.05 in the fifth quantile, indicating that the optimal resource allocation would be allocated 72–80 percent of SSP household budget in the first to third quantile. These findings provide empirical support for Pakistan’s financing for health and are important in optimizing the design and implementation of the SSP Plan.

While the LDMR phase partly points to the non-linear efficiency of health investments: increased profits: increased profits: increased revenues for each additional investment block lead to more significant improvements in health (first to second quantile); Maximum efficiency: achieving maximum productivity; The next division will receive equal profit (q3); Decline in revenue: additional investments yield lower profit margins (fourth quantile); Negative benefit: additional resources will reduce overall efficiency (q5).

## 4 Discussion

Our findings provide robust empirical evidence that Pakistan’s Sehat Sahulat Program (SSP) along with other government related program exhibits diminishing marginal returns across wealth quintiles, with significantly greater impacts on child nutrition outcomes among poorer households. This result is consistent with economic theory and confirms new evidence for other low- and middle-income countries implementing similar health financing programs (51–54). However, it is important to acknowledge the limitations of our cross-sectional data. While IV methods help address endogeneity, causal claims should be interpreted with caution. Longitudinal or experimental data would strengthen causal inference regarding SSP’s impact.

The steep socio-economic gradient in the effectiveness of SSP reflects the results of a similar conditional cash transfer scheme in South Asia. Like the Rashtriya Swasthya Bima Yojana (RSBY) in India, SSP showed a particularly strong influence on one fifth of the minimum wealth (55–57). Nevertheless, our study reinforces this document by accurately quantifying marginal returns (Q3) declines—a new contribution that has direct relevance to policy.

In households during the first to third quarter SSP’s performance against private insurance supported the argument that public funding of health insurance in limited resources (58, 59). This finding challenges assumptions about the efficiency of the private sector and is consistent with the results of Thailand’s universal coverage plan (60, 61). The 72 percent optimal distribution thresholds we have set provide specific guidance to the planned targets that will improve the cost-effectiveness of the SSP program. Our findings on the corrective effects of sanitation reinforced the nexus between WASH—nutrition (62).

Quantile regression analysis revealed an important insight into the relationship between health interventions and diarrhea morbidity. The marginal benefits of sanitation were non-linear, and flexibility decreased from the first to the fifth quantile, indicating that the effectiveness of wealthier households is declining. The negative flexibility of the top wealth quintile means that additional investment in sanitation has produced little or even backward results, when basic coverage is already high. These findings are consistent with available evidence that health interventions bring disproportionate benefits to low-income groups with weak baselines and high health risks. Past studies also indicate that returns on investment in sanitation will decline as sanitation coverage increases, especially in wealthier households that have improved sanitation (63, 64). This model suggests that health for all projects may be less cost-effective than targeted approaches focused on underserved communities. Research also suggests that the health effects of sanitation go beyond infrastructure, and that complementary behavioral and educational components are needed to maximize benefits (65, 66).

Moreover, the incidence of diarrhea fell sharply from 31.5 percent of the poorest quintile to 12.7 percent of the richest quintile. The diarrhea epidemic has increased the demand for equity-oriented policies, which should give priority to vulnerable groups while avoiding the inefficient allocation of resources to populations that already have adequate sanitation (67, 68). The observed gradient in diarrhea rates, from the poorest quintile to the richest quintile, highlights the long-term effects of socio-economic determinants on children’s health. The response has been consistent in the case of Pakistan, where qualitative and quantitative studies indicate that rural and marginalized mothers face significant challenges in managing childhood diseases, such as diarrhea, and are often constrained by lack of resources, awareness and access to adequate water and sanitation (69–72). The acute gradient of diarrhea rates underscores the strong role of socio-economic status and geography in shaping children’s health outcomes, a finding strongly supported by national data on malnutrition (73). These obstacles are compounded by economic pressures and poor households, which are key predictors of malnutrition and poor health, as illustrated by the CVID 19 (74) blockade. Furthermore, the nutritional challenges are complex, as studies in similar environments emphasize the critical role of lack of food diversity in causing malnutrition and reveal the increasing double burden of malnutrition in poor communities (75, 76). The interactions between poverty, and malnutrition in vulnerable communities lead to a vicious cycle of high rates of diarrheal diseases and malnutrition (53, 77). Therefore, a growing consensus, that is, case-specific, basing and tailored toward the financial constraints of interventions—such as maternal awareness resolution and targeted health infrastructure interventions and social security packages such as health insurance (78–83)—is critical to breaking that cycle, and is underserved in malnutrition and diarrheal diseases. Health improvements have led to more than standardized methods, increasing the need for targeted interventions in underserved communities.

This has given rise to many theoretical meanings. First, we examined the health insurance LDMR report that economic principles may be more pervasive on health interventions than previously thought. Second, the skewed function of health production that we have outlined is important for making decisions about health investment. Thirdly, our approach provides a replicable framework for assessing other social protection projects.

From a policy viewpoint, these discoveries emerged at a critical juncture in Pakistan where Pakistan is considering expanding the SSP Initiative. The evidence strongly supports a focus on poverty reduction targets rather than a universal coverage approach. Our key proposals (maximum distribution of Q1-Q3, progressive in Q4, and reduction and minimum in Q5) provide a practical roadmap for optimizing the use of limited resources. This study has contributed to the debate on health financing by demonstrating how economic principles (i.e., LDMR) guide equitable health systems. Our findings indicate that achieving the sustainable development goals for child nutrition requires not only scaling up health coverage, but also strategically targeting interventions with the greatest marginal returns.

## 5 Conclusion

This study, providing, for the first time, empirical evidence of diminishing marginal returns to health insurance effectiveness in Pakistan, indicates that the health improvement achieved by Sehat Sahulat Program (SSP) on the health of poor households was much greater than that of rich households. Using multi-model analysis techniques—probability models (Logit and Probit), instrumental variable for endogeneity tests related to health insurance (IV-Probit) and quantile regression (IV-QR) for heterogeneity by wealth level. Study found that SSP gains fell sharply after the middle wealth quintile (Q3), and the impact on nutritional outcomes of children from wealthier households (Q5) were negligible. These findings confirm that Law of Diminishing Marginal Returns (LDMR) is a key framework for assessing health financing policies in low- and middle-income countries, particularly Pakistan. Furthermore, IV-Probit results indicate that health insurance coverage reduces stunting by 92 percent and diarrhea by 98 percent, respectively.

Our analysis indicates that the reallocation of 72 percent of SSP resources to the poorest families (first to third quantile) would achieve maximum cost-effectiveness while maintaining financial sustainability. This targeted approach not only leads to more efficient planning, but also contributes to equitable health outcomes by prioritizing those most in need. The accuracy of the study methodology—in particular, the use of IV quantile regression to explain wealth-based heterogeneity—reinforces causation and provides policymakers with operational thresholds for improving health insurance coverage.

These discoveries had a direct impact on the ongoing expansion of SSP in Pakistan. The pattern of diminishing returns suggests that the best goals of the SSP pointing should be prioritized for households in the first to third quantiles, which together account for about 72 percent of potential health benefits. Resources can be distributed gradually, with the highest subsidy rate (85–90 percent) for the first quantile, medium support (75–80 percent) for the second quantile, and basic coverage (60–65 percent) for the third quantile. For households in the fourth to fifth quantiles, it may be more appropriate to replace the intervention or cost-sharing mechanisms, given the marginal returns observed. This evidence-based approach to target setting would maximize the health benefits of the SPP expenditure per rupee, while maintaining the sustainability of the program. Future research should explore how complementary interventions (such as complementary feeding and improved health conditions) can further enhance the impact of interventions in marginalized groups. By linking microeconomic theory to health policies, the study highlights the importance of achieving the evidence-based goal of universal health coverage without increasing inequality.

### 5.1 Limitations of this study

Although this study provides new insights into the diminishing returns of health insurance benefits, some limitations should be recognized. To begin with, analysis relies on cross-section data based on PDHS (2017–18), reducing causal reasoning and IV-QR and IV-Probit. Second, due to data constraints, key variables of socio-economic linkages, such as household food security, maternal nutritional diversity and seasonal income fluctuation, which are known to affect child stunting independently of insurance, are excluded. Furthermore, research has reduced coordinated variables by avoiding excessive interpretation or results, and a combination of understanding of research variables. Although these omissions may affect accuracy, the quintile of our wealth and concern for marginal (but not absolute) effects have mitigated the deviations. But with regard to descriptive statistics, we have already provided possible socio-economic variables. Finally, the exclusion of other variables related to socio-economic cohesion is due to the assessment of the relative income of groups of wealth, not to the absolute determinants of stunting, and to avoid common linear problems. Future vertical studies should incorporate these common variables to further improve objectives.

## Data Availability

The original contributions presented in the study are included in the article/supplementary material, further inquiries can be directed to the corresponding authors.
